# PD-L1 has a heterogeneous and dynamic expression in gastric cancer with implications for immunoPET

**DOI:** 10.3389/fimmu.2024.1405485

**Published:** 2024-06-10

**Authors:** Dina Ibrahim, Cristina Simó, Emma L. Brown, Shayla Shmuel, Sandeep Surendra Panikar, Alex Benton, Rachel DeWeerd, Farrokh Dehdashti, Haeseong Park, Patrícia M. R. Pereira

**Affiliations:** ^1^ Department of Radiology, Mallinckrodt Institute of Radiology, Washington University School of Medicine, St. Louis, MO, United States; ^2^ Cancer Biology Graduate Program, Washington University School of Medicine, St. Louis, MO, United States; ^3^ Gastrointestinal Cancer Center, Center for Cancer Therapeutic Innovation, Dana-Farber Cancer Institute, Harvard Medical School, Boston, MA, United States

**Keywords:** PD-L1, *N*-glycosylation, gastric tumors, immunoPET, avelumab, atezolizumab

## Abstract

**Introduction:**

This study aimed to investigate the dynamics of programmed death-ligand 1 (PD-L1) expression, spatial heterogeneity, and binding affinity of FDA-approved anti-PD-L1 antibodies (avelumab and atezolizumab) in gastric cancer. Additionally, we determined how PD-L1 glycosylation impacts antibody accumulation in gastric cancer cells.

**Methods:**

Dynamic PD-L1 expression was examined in NCIN87 gastric cancer cells. Comparative binding studies of avelumab and atezolizumab were conducted in gastric cancer models, both *in vitro* and *in vivo*. Antibody uptake in tumors was visualized through positron emission tomography (PET) imaging. PD-L1 glycosylation status was determined via Western blot analyses before and after PNGase F treatment.

**Results:**

Consistent findings revealed time-dependent PD-L1 induction in NCIN87 gastric cancer cells and spatial heterogeneity in tumors, as shown by PET imaging and immunofluorescence. Avelumab displayed superior binding affinity to NCIN87 cells compared to atezolizumab, confirmed by *in vivo* PET imaging and *ex vivo* biodistribution analyses. Notably, PD-L1 glycosylation at approximately 50 kDa was observed, with PNGase F treatment inducing a shift to 35 kDa in molecular weight. Tissue samples from patient-derived xenografts (PDXs) validated the presence of both glycosylated and deglycosylated PD-L1 (degPD-L1) forms in gastric cancer. Immunofluorescence microscopy and binding assays demonstrated enhanced avelumab binding post-deglycosylation.

**Discussion:**

This study provides an understanding of dynamic and spatially heterogeneous PD-L1 expression in gastric cancer. Anti-PD-L1 immunoPET was able to visualize gastric tumors, and PD-L1 glycosylation has significant implications for antibody recognition. These insights contribute to demonstrating the complexities of PD-L1 in gastric cancer, holding relevance for refining PD-L1 imaging-based approaches.

## 1 Introduction

Programmed cell death-1 (PD-1) and its ligand, PD-L1, belong to the family of immune checkpoint proteins that play a crucial role in regulating T cell tolerance and immune evasion in cancer (1, 2). PD-L1 on tumor cells interacts with its cognate receptor, PD-1, on T cells to suppress activation, expansion, and effector functions of antigen-specific CD8^+^ T cells (1, 2).

Immune checkpoint inhibitors (ICI) targeting PD-1 and PD-L1 have revolutionized the treatment of various cancers. In gastric cancer, PD-1 inhibitors, including pembrolizumab and nivolumab, have established benefits in the treatment of advanced diseases (3). However, the clinical benefit of PD-1/PD-L1 blockade is not universal, and PD-L1 expression remains the only clinically relevant predictive biomarker despite its limitations. High levels of tumor heterogeneity in PD-L1 expression in gastric cancer, along with variability across different assays, are some of the key limitations of utilizing tissue PD-L1 expression as the sole biomarker to identify patients that may benefit from ICI in gastric cancer. Furthermore, not all ICIs have resulted in improved outcomes for patients with gastric cancer. Clinical trials of ICI in later lines of therapy or those using PD-L1 inhibitors have less impactful results (4–6). These efforts highlight the need for a better assessment of PD-L1 status that may reflect both spatial and temporal heterogeneity of its expression.

Several biomarkers, ranging from tumor mutation burden and PD-L1 expression to microsatellite instability and Epstein–Barr virus infection status, have been proposed as potential indicators for identifying susceptibility to PD-1/PD-L1 inhibitors in gastric cancer (7, 8). However, the outcomes of several clinical trials utilizing these biomarkers at an individual level exhibit inconsistency and, in some instances, even contradiction (9–11). This lack of consistency in findings underscores the absence of a singular biomarker capable of adequately stratifying patients, not only within the context of gastric cancer but also across other tumor types. Clinical and preclinical positron-emission tomography (PET) studies using radiolabeled anti-PD-L1 antibodies have successfully demonstrated non-invasive imaging of PD-L1 expression in tumors with potential for clinical response prediction to ICI (12–16). While PD-L1-targeting PET approaches hold the potential to provide valuable insights into the assessment of heterogeneity and PD-L1 status throughout the whole body and predict response to ICI, their applicability in the context of gastric cancer imaging remains uncertain.

PET imaging with radiolabeled anti-PD-L1 antibodies atezolizumab and avelumab presents a unique opportunity to provide information about the tumor immune infiltrate and its response to therapy. However, the complex spatial and temporal dynamics of PD-L1 expression, including its dynamic glycosylation patterns (*e.g.* addition of carbohydrate molecules, glycans, to the asparagine residue at the *N*-terminal domain of the protein), present a challenge for consistent antibody binding to tumors (17, 18). PD-L1 glycosylation influences the binding of anti-PD-L1 antibodies to tumors, which could hinder the application of PET imaging. Indeed, the removal of *N*-linked glycosylation has been suggested as a strategy to enhance anti-PD-L1 antibody binding to tumors and therefore improve ICI efficacy (19, 20). Understanding the impact of PD-L1 glycosylation on antibody interactions is essential for refining the precision and reliability of immunoPET imaging in gastric cancer.

This preclinical study aimed to assess the feasibility of anti-PD-L1 antibodies, avelumab and atezolizumab in PET imaging of gastric cancer. Furthermore, it sought to investigate how the dynamic expression of PD-L1 in gastric cancer, influenced by its glycosylation patterns may impact the binding of these antibodies to gastric cancer cells.

## 2 Materials and methods

### 2.1 Cell culture and interferon-gamma treatments

The NCIN87 human gastric cancer cells used in this study were cultured at 37°C under 5% CO_2_ and 99% humidity. NCIN87 cells were obtained from the American Type Culture Collection (Manassas, USA), and were used within 20 passages. NCIN87 cells were routinely screened for possible mycoplasma contamination. NCIN87 cells were maintained in Roswell Park Memorial Institute 1640 (RPMI-1640) growth medium supplemented with 10% (v/v) fetal bovine serum, 2 mM L-glutamine, 10 mM 4-(2-hydroxyethyl)-1-piperazineethanesulfonic acid, 1 mM sodium pyruvate, 4.5 g/L solution of glucose, 1.5 g/L sodium bicarbonate, and 100 unit/mL of an antibiotic mixture containing penicillin and streptomycin.

To induce PD-L1 expression *in vitro*, NCIN87 cancer cells were incubated with recombinant human interferon-gamma (IFN-γ, Roche Diagnostics, Germany) at 0.5 ng per one million of NCIN87 cancer cells. The culture media were replenished daily with a fresh addition of IFN-γ.

### 2.2 Deglycosylation using PNGase F

In experiments involving PNGase F treatments, NCIN87 cell lysates were prepared in radioimmunoprecipitation (RIPA) buffer: 150 mM NaCl, 50 mM Tris-HCl, pH 7.5, 5 mM egtazic acid, 1% (v/v) Triton X-100, 0.5% (w/v) sodium deoxycholate, 0.1% (w/v) sodium dodecyl sulfate, 2 mM phenylmethanesulfonyl, 2 mM iodoacetamide, and 1× protease inhibitor cocktail [C852A33; Roche].

Tumor lysates were prepared using a tissue homogenizer 150 (Fisher brand) in RIPA buffer.

Cell and tumor lysates were then centrifuged at 18,000*×g* for 16 min at 4°C. Next, the deglycosylation of PD-L1 in both cell and tumor lysates was performed using PNGase F (New England BioLabs, Ipswich, MA, USA) as described by the manufacturer. Briefly, 1 µL of denaturing buffer 10X, 2 µL of GlycoBuffer 10X, and 2 µL of 10% Nonidet P-40 were added to 40 µg of protein lysates to make up a 20 µL total reaction volume. The mixture was incubated at 37°C overnight without or with 1 µL of PNGase F (final glycerol concentration equal to 5% v/v). The mixture was then denatured in Laemmli buffer and heated at 95°C for 7 min or in a water bath at 37°C for 1.5 h. The samples were subsequently mixed using a vortex and then cooled to room temperature in preparation for Western blot analyses.

### 2.3 Western blot of whole, cell-surface, and internalized protein extracts

Western blots targeting cell-surface PD-L1 were performed using biotin pull-down assays. NCIN87 cells, pre-treated with IFN-γ (0.5 ng per 1 million), underwent two washes with ice-cold phosphate buffered saline (PBS) containing 0.5 mM magnesium chloride (MgCl_2_) and 1 mM calcium chloride (CaCl_2_). Subsequently, NCIN87 cells were incubated with 0.5 mg/mL EZ-LINK Sulfo-Biotin (Thermo Fisher Scientific) for 30 minutes at 4°C with gentle rotation. The reaction was stopped by two washes with 100 mM glycine (Thermo Fisher Scientific) in PBS containing 0.5 mM MgCl_2_ and 1 mM CaCl_2_. After scraping the cells in RIPA, lysates were centrifuged at 18,000*×g* for 16 minutes at 4°C, and the collected supernatants were assayed for protein concentration using the Pierce Bicinchoninic acid (BCA) Protein Assay Kit (Thermo Fisher Scientific). A 500 μL volume of RIPA buffer, containing an equal amount of proteins, was incubated with NeutrAvidin Agarose Resins (Thermo Fisher Scientific) overnight at 4°C with gentle rotation. The resins were washed three times with RIPA buffer before suspension in Laemmli buffer.

To collect internalized PD-L1, cell surface-biotinylation was first performed as described above. Next, the endocytosis of membrane proteins was promoted by the addition of 1 μM avelumab in complete media at room temperature for 1 h. The non-internalized cell-surface biotinylated proteins were removed by incubating cells with 50 mM Tris-HCl pH 8.7 [containing 20 mM dithiothreitol (DTT), 100 mM NaCl, 2.5 mM CaCl2] for 20 min at 4°C. After collecting protein lysates in RIPA buffer, the biotinylated internalized proteins were collected in NeutrAvidin agarose resins and Western blot analyses was performed as described above.

Protein extracts from control or treated NCIN87 cells were denatured at 96°C for 7 min and cooled to room temperature. Proteins were separated in 4–12% Bis-Tris gels (Invitrogen) and transferred onto nitrocellulose or PVDF membranes using iBlot transfer stacks (Invitrogen). The membranes were blocked with 5% (w/v) non-fat milk (Bio-RAD) or bovine serum albumin (BSA, Sigma) in Tris-buffered saline-Tween (TBS-T, Cell Signaling Technology) at room temperature for 1 h and incubated with 1:500 rabbit anti-PD-L1 antibody (E1L3N, Cell Signaling) or 1:10,000 mouse anti-β-actin (A1978; Sigma) overnight at 4°C. Following three washing steps, the membranes were incubated with IRDye 680CW anti-rabbit (925–32211) or IRDye 800CW anti-mouse (925–32210) IgG antibodies (LI-COR Biosciences) at a dilution of 1:10,000. After washing, the membranes were imaged on an Odyssey Infrared Imaging System (LI-COR Biosciences). Densiometric analysis of the respective bands was performed using ImageJ/FIJI (NIH, USA; https://imagej.net/Fiji).

Whole-protein extracts from mouse organs (skin, muscle, bone marrow, spleen, stomach, large intestine, small intestine, heart, pancreas, kidney, liver, lung, and brain) were prepared after tissue homogenization in RIPA buffer and protein separation as described above. Membranes were probed using the following mouse-reactive primary antibody: 1:1,000 rabbit anti-mouse-PD-L1 (D4H1Z; Cell Signaling). The revert 700 total protein stain (LI-COR Biosciences) was used as loading control. Membranes were imaged on an Odyssey Infrared Imaging System (LI-COR Biosciences) and quantified using the Empiria Studio Software.

### 2.4 Immunohistochemistry and immunofluorescence in tumor tissues

PD-L1 immunohistochemistry was performed by the Laboratory of Comparative Pathology at Memorial Sloan Kettering Cancer Center.

For immunofluorescence studies involving fluorescently labeled avelumab and atezolizumab, antibodies were first labeled with Alexa Fluor 488 at a molar ratio of 3 fluorophores per antibody at 37°C in PBS buffer at pH 8.8 for 1 h. The fluorescently labeled antibody conjugates were purified via size exclusion chromatography (PD-10 column; GE Healthcare) and concentrated using 50 kDa cutoff Amicon filters. De-identified gastric PDX tissues were obtained from the Antitumor Assessment Core at Memorial Sloan Kettering Cancer Center through a material transfer agreement (MTA-Out00001384). Paraffin-embedded PDX tissues were sectioned to 10 µm thickness. Before staining, sections were deparaffinized using Neo-Clear and decreasing alcohol concentrations (100% ethanol for 1 min, 96% v/v ethanol for 1 min, 70% v/v ethanol for 1 min) followed by 2 washes in deionized water for 1 min each. Following deparaffinization, sections were permeabilized for 30 min using 0.25% (v/v) Triton X-100 (Alfa Aesar) prepared in PBS (0.022% NaN3 w/v, BSA 0.02% w/v), before blocking with 10% (v/v) goat serum for 30 min at room temperature. Sections were then incubated with 200 nM of Alexa 488-labeled avelumab or atezolizumab overnight at 4°C in a humidified chamber. After washing, the tissue sections were stained with DAPI and mounted using Dako mounting medium (Agilent) and imaged on EVOS M5000 Imaging microscope (Invitrogen).

For tumor tissue deglycosylation, PDX and NCIN87 tissues were deparaffinized as described above. Tissues sections were incubated with 1X glycoprotein denaturing buffer at room temperature for 3 h in a humidified chamber. After washing with PBS, tissue sections were treated with or without the presence of PNGase F (5%) containing PBS overnight at room temperature in a humidified chamber. Following deglycosylation, sections were permeabilized as described before. Tissues sections were then incubated with 200 nM of Alexa 488-labeled avelumab at room temperature for 5 h in a humidified chamber. After washing, the tissue sections were mounted using Dako mounting medium (Agilent) and imaged on EVOS M5000 Imaging microscope (Invitrogen).

### 2.5 Immunofluorescence in NCIN87 gastric cancer cells

Immunofluorescence (IF) studies were performed in NCIN87 cells with or without PNGase F treatment (21, 22). Avelumab was initially conjugated with Alexa Fluor 488 following the procedure described above.

NCIN87 cells, treated with human IFN-γ for 72 h (0.5 ng per 1 million), were cultured on pre-coated poly-L-lysine coverslips. The cells were washed and incubated with PNGase F (5,000 units/mL) in serum-free culture media at 37°C for 6 h. For control slides, the cells were kept in serum-free culture media without PNGase F. The cells were then washed with PBS containing 0.1% w/v BSA and incubated with Alexa-488 fluorescently labeled avelumab (100 nM) at 4°C for 30 min then 37°C for 1.5 h. NCIN87 cells were then washed 3 times with PBS and fixed in 4% (v/v) paraformaldehyde (PFA), and subsequently washed three times with PBS. Coverslips were mounted using Dako mounting media (Agilent), and fluorescent images were captured using the EVOS M5000 Imaging microscope (Invitrogen).

### 2.6 Radiolabeling of avelumab and atezolizumab

Zirconium-89 was obtained from the Washington University School of Medicine Cyclotron Facility. To prepare zirconium-89 (^89^Zr)-labeled avelumab or atezolizumab, the antibody was first conjugated with the chelate *p*-isothiocyanatobenzyl-desferrioxamine (DFO-Bz-NCS; Macrocyclics, B-705) at a ratio of 5:1 (DFO:antibody) at 37°C for 1 h with slow agitation in PBS at a pH of 8.8. The unconjugated DFO was removed after the reaction using a size exclusion column (PD-10; GE Healthcare) and the antibody solutions were concentrated in chelex-PBS (pH 7.7) using Amicon filters with a 50 kDa cutoff filter. Matrix-assisted laser desorption/ionization-time of flight (MALDI-TOF) mass spectrometry of the antibody conjugates was performed at the Alberta Proteomics and Mass Spectrometry Facility at the University of Alberta in Canada, to determine the number of conjugates per antibody.

The antibody-DFO conjugates were then radiolabeled with zirconium-89 at 4.36 μCi/μg of antibody-DFO in a chelex-PBS solution at pH 7.4. Radiolabeled antibody products were purified and concentrated using PD-10 columns and Amicon filters with a 50 kDa cutoff as described above. The radiochemical purity (RCP) of the radiolabeled conjugates used *in vitro* and *in vivo* was determined by instant thin-layer chromatography using 50 mM ethylenediaminetetraacetic acid (EDTA) pH 5.5 as mobile phase. The RCP of the conjugates used for *in vitro* and *in vivo* studies was 99%. The radiochemical conversion yields ranged from 95 to 98%, and the molar activity was 23.01 MBq/nmol. The immunoreactivities were above 90%. In all experiments where avelumab and atezolizumab were compared *in vitro* and *in vivo*, they were simultaneously conjugated and radiolabeled with zirconium-89 from the same batch production.

### 2.7 Binding, blocking, and serum stability assays

NCIN87 cells (1 million) treated with IFN-γ were incubated with 0.037 MBq of ^89^Zr-labeled avelumab or atezolizumab for 1 h at 4°C. Blocking experiments were performed by incubating cells with the radiolabeled antibody in the presence of a 100-fold excess (22 μg) of unlabeled DFO-avelumab or DFO-atezolizumab. The gamma counter 2480 Wizard (PerkinElmer) was utilized to measure the radioactivity in both the supernatants and the NCIN87 cell pellet, and immunoreactivity was determined by dividing the radioactivity of the cell pellet by the total radioactivity in the cell pellet and washing fractions.

For serum stability assays, 0.0185 MBq of [^89^Zr]Zr-DFO-avelumab or [^89^Zr]Zr-DFO-atezolizumab (0.11 μg) were incubated in 1 mL of human serum (Sigma) at 37°C for a duration of 5 days. Daily evaluations of radiochemical purity were conducted using instant thin-layer chromatography with 50 mM ethylenediaminetetraacetic acid as the mobile phase.

### 2.8 Saturation binding assay

NCIN87 cells incubated with IFN-γ were exposed to varying concentrations of [^89^Zr]Zr-DFO-avelumab or [^89^Zr]Zr-DFO-atezolizumab (0 to 256 nM) in PBS (pH 7.5) containing 1% (w/v) human serum albumin (Sigma) and 1% (w/v) sodium azide (Acros Organics). The incubation was performed for 2 h at 4°C. Following cell incubation with the radiolabeled antibodies, unbound radioactivity was removed, and the cells were washed three times with PBS. To measure the total cell-bound radioactivity, the cells were solubilized in 100 mM sodium hydroxide and collected into scintillation vials. The radioactivity associated with the cells was quantified using a gamma counter (2480 Wizard, PerkinElmer) that was previously calibrated for zirconium-89. The obtained data for total binding were plotted against the concentration of [^89^Zr]Zr-DFO-avelumab or [^89^Zr]Zr-DFO-atezolizumab added to the cells. To determine B_max_ (the maximum binding capacity), the data were fitted using a 1-site binding model through nonlinear regression analysis performed in GraphPad Prism 7.00. The nonspecific binding component was subtracted from the total binding using a non-specific ^89^Zr-labeled IgG to generate specific binding curves.

### 2.9 Tumor xenografts

Female nude mice at the age of 6–8 weeks were purchased from Charles River Laboratories. The mice were inoculated on the right flank with 5 million NCIN87 cells in a 100 μL cell suspension of a 1:1 (v:v) mixture of medium with reconstituted basement membrane (BD Matrigel, BD Biosciences). All animal experiments were conducted following guidelines approved by Washington University School of Medicine’s Research Animal Resource Center and Institutional Animal Care and Use Committee. Animals were housed in type II polycarbonate cages and provided with a sterilized diet and water *ad libitum*. Mice were maintained under 12 h dark/light cycles, at 22°C and 60% relative humidity.

External caliper measurements were performed to estimate the volume of tumors (V, mm^3^). The longest axis (a, mm) and the short axis (b, mm) are perpendicular to each other, and the tumors were assumed to have a spherical shape. Tumor volumes were calculated using the equation V= (4π/3) x (α/2)^2^ x (b/2). V is tumor volume (mm^3^), α is the longest axis (mm), and b is the axis perpendicular to the longest axis (mm). When the tumor volume reached approximately 200 mm^3^, mice were randomly divided into groups (n = 5 mice per group for biodistribution and n = 4 mice per group for PET imaging).

### 2.10 Hematoxylin and eosin and PD-L1 staining

NCIN87 tumors, lymph nodes, and spleen tissues that were previously formalin-fixed and paraffin-embedded were sectioned to 10 µm thickness. Before staining, sections were deparaffinized using Neo-Clear and decreasing alcohol concentrations (100% v/v ethanol for 1 min, 96% v/v ethanol for 1 min, 70% v/v ethanol for 1 min) followed by two washes with deionized water for 1 min each. For H&E staining, the tissue sections were then stained according to the manufacturer’s instructions (Vector laboratories).

For IF staining of tumors, lymph nodes, and spleen tissues, following deparaffinization as described above, sections were permeabilized for 30 min using 0.25% (v/v) Triton X-100 (Alfa Aesar) prepared in PBS (BSA 0.02% w/v), before blocking with 10% v/v goat serum for 30 min at room temperature. Sections were then incubated with 1:100 rabbit anti-PD-L1 antibody (E1L3N, Cell Signaling), 1:100 rabbit anti-CD11b antibody (ab1333357, Abcam), or 1:100 mouse anti-cytokeratin (ab27988, Abcam) prepared in PBS containing 0.02% (w/v) BSA overnight at 4°C in a humidified chamber. After washing, the tissue sections were incubated with 1:250 of Alexa568 or Alexa488-conjugated secondary antibodies per the manufacturer’s instructions (Thermo Fisher Scientific). Once staining was completed, tissue sections were mounted using Dako mounting medium (Agilent) and imaged on EVOS M5000 Imaging microscope (Invitrogen).

### 2.11 PET/CT imaging and acute biodistribution studies

Imaging experiments were performed using a Mediso nanoScan PET/CT scanner (Mediso). To ensure adequate anesthesia, mice were administered 2–3% isoflurane (Baxter Healthcare) in an oxygen gas mixture by inhalation 10 minutes before acquiring PET images. Mice were kept under 2% isoflurane in an oxygen gas mixture during PET/CT scan acquisitions. PET data for each group (n = 4) were obtained at different time points (24, 48, and 72 h) after intravenous injection of the ^89^Zr-labeled antibody (3.3 – 3.7 MBq, 21–23 µg of [^89^Zr]Zr-DFO antibody plus 29–27 µg of unlabeled antibody-DFO to make it a total of 50 µg administered antibody). Images were analyzed using 3D Slicer software (version 5.0.3, a free and open source software https://www.slicer.org/).

Biodistribution studies were conducted at 24, 48, and 72 h post-injection of the radiolabeled antibody. Additional biodistribution studies were performed at 48 h post-injection of [^89^Zr]Zr-DFO-avelumab injected in the presence of unlabeled DFO-avelumab (25X, 1.25 mg). Following mice euthanasia by controlled carbon dioxide overdose followed by cervical dislocation, organs were collected, and radioactivity was assessed using a gamma counter (2480 Wizard, PerkinElmer). The radioactivity associated with each organ was quantified as a percentage of the injected dose per gram of organ (% ID/g), and tumor-to-organ ratios.

### 2.12 Statistical analyses

Statistical analyses were performed using Prism V.9.0 (GraphPad Soft-ware Inc, San Diego, Canada) and represented as the mean ± SD. Student *t* tests were run assuming unequal variances. For blocking study, a two-way ANOVA Šídák’s multiple comparisons test was performed. A *p* value ≤ 0.05 was considered statistically significant.

## 3 Results

### 3.1 PD-L1 expression in gastric tumors

**Figure 1:**
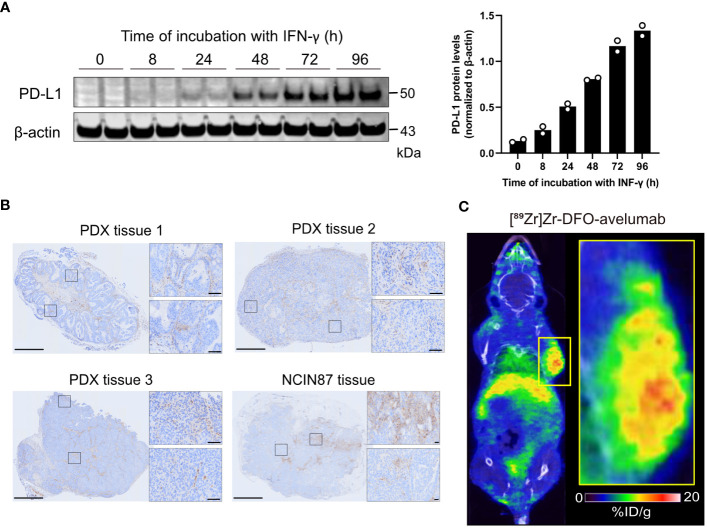
PD-L1 expression in gastric cancer cells and tumor tissues. **(A)** Western blot of whole NCIN87 cell lysates after cells’ incubation with INF-γ (0.5 ng IFN-γ per 1 million of NCIN87 cancer cells) during 0, 8, 24, 48, 72, 96 (h). The quantification of PD-L1 protein levels was normalized to β-actin at each time point in duplicate. **(B)** PD-L1 immunohistochemistry in gastric PDXs and NCIN87 gastric tumor tissues. Scale bar, 1 mm and 50 µm. **(C)** Representative whole-body PET/CT images acquired 48 h post tail vein injection of [^89^Zr]Zr-DFO-avelumab in mice bearing NCIN87 xenografts.

PD-L1 expression is regulated by a wide complex interplay of biological factors, including inflammatory cytokines such as INF-γ. To induce PD-L1 expression *in vitro*, NCIN87 gastric cancer cells were incubated with INF-γ for 8, 24, 48, 72, or 96 h. INF-γ induced PD-L1 expression in NCIN87 cells in a time-dependent manner (**Figure 1A**, **Supplementary Figure 1**). Additional PD-L1 IHC in NCIN87 tumor tissues revealed variable and heterogeneous expression, similar to that of gastric cancer tissues from patient-derived xenografts (**Figure 1B**, **Supplementary Figure 2**). Consistent with prior studies in other cancer cell lines (23, 24), immunofluorescence studies demonstrated PD-L1 staining at the membrane and within the nucleus of NCIN87 tumors (**Supplementary Figure 3**).

Next, we assessed PD-L1 distribution in NCIN87 tumors using PET imaging with the anti-PD-L1 antibody avelumab labeled with the positron emitter zirconium-89 ([^89^Zr]Zr-DFO-avelumab). We observed intra-tumoral heterogeneity of [^89^Zr]Zr-DFO-avelumab uptake in NCIN87 xenografts (**Figure 1C**).

Overall, our observations highlight the spatially heterogeneous expression of PD-L1 in NCIN87 gastric cancer.

### 3.2 Avelumab has higher binding for gastric cancer cells when compared with atezolizumab

Avelumab and atezolizumab are two FDA-approved anti-PD-L1 antibodies. In contrast to atezolizumab, which was designed to minimize Fc-mediated effector functions, avelumab maintains the capability to induce antibody-dependent cellular cytotoxicity and complement-dependent cytotoxicity (25). Other studies have shown that avelumab has a superior binding affinity to PD-L1 compared to atezolizumab (26, 27). Given the differences in avelumab versus atezolizumab binding to tumors, we sought to determine the binding of these two antibodies to NCIN87 gastric cancer cells following INF-γ stimulation. First, we prepared the immunoPET probes by conjugating avelumab or atezolizumab with the DFO chelator (**Supplementary Figure 4**) and then radiolabeling with zirconium-89. The specific activity of the acquired [^89^Zr]Zr-DFO-antibody (4.36 μCi/μg) aligned with what was previously reported (28). [^89^Zr]Zr-DFO-avelumab and [^89^Zr]Zr-DFO-atezolizumab stability was above 90% after incubation in human serum for a period of 5 days (**Supplementary Figures 5, 6**).

**Figure 2:**
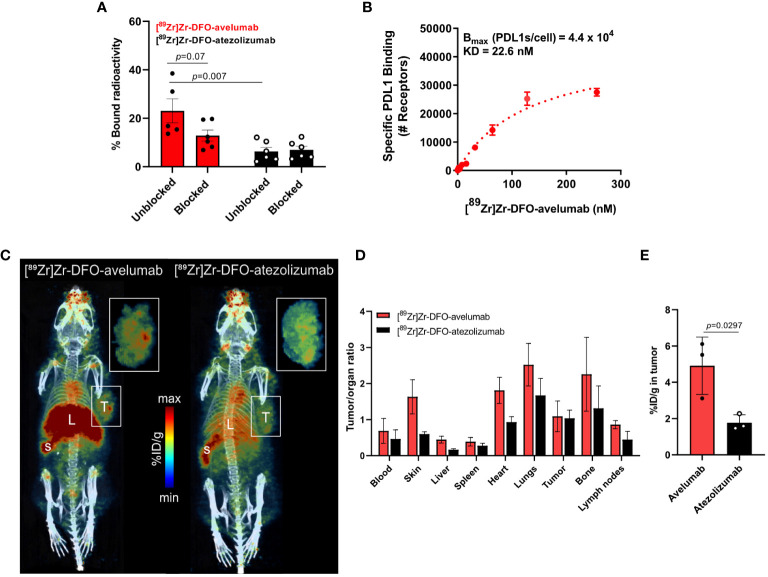
Avelumab uptake is higher than atezolizumab in NCIN87 gastric tumors. **(A, B)** *In vitro* binding and saturation binding assay of [^89^Zr]Zr-DFO-avelumab or [^89^Zr]Zr-DFO-atezolizumab in NCIN87 cells treated with INF-γ for 72 (h). The radiolabeled anti-PD-L1 antibody was added to the wells in increasing concentrations (0 to 256 nM). The cold block DFO-anti-PD-L1 antibody was added in a 100-fold excess prior to the radiolabeled PD-L1 antibody. Data are presented as mean ± S.E.M., n = 5. Statistical analysis was performed using a Student *t* test, significance was considered for *P*<0.05. **(C)** Representative PET/CT images of *nu/nu* mice bearing NCIN87 tumors at 24 h post-injection of [^89^Zr]Zr-DFO-Avelumab (left) and [^89^Zr]Zr-DFO-Atezolizumab (right). Scale bar represents % ID/g, percentage of injected dose per gram. S-spleen, L-liver, T-tumor. Pop-out shows tumor uptake. **(D, E)** Tumor to organ ratios of the mean [^89^Zr]Zr-DFO-avelumab and [^89^Zr]Zr-DFO-atezolizumab uptake at 24 h post-injection. Tumor uptake of [^89^Zr]Zr-DFO-avelumab and [^89^Zr]Zr-DFO-atezolizumab at 24 h post-injection.

[^89^Zr]Zr-DFO-avelumab binding to NCIN87 gastric cancer cells was higher (*p*=0.007) compared to [^89^Zr]Zr-DFO-atezolizumab (**Figure 2A**). Additional binding studies demonstrated a higher (*p*=0.07) percentage of bound radioactivity in [^89^Zr]Zr-DFO-avelumab group versus the block group (cells incubated with [^89^Zr]Zr-DFO-avelumab in the presence of 100-fold of unlabeled avelumab).

Competitive radioligand saturation-binding assays with [^89^Zr]Zr-DFO-avelumab, demonstrated that NCIN87 cancer cells express 44,000 PD-L1 per cell (**Figure 2B**), which is higher than previous studies in the gastric cancer cell line MKN45 (<2,200 PD-L1 per cell) (28). Altogether, these results indicate that avelumab has a higher affinity for PD-L1 in gastric cancer cells compared to atezolizumab.

### 3.3 [^89^Zr]Zr-DFO-avelumab localizes in subcutaneous gastric tumors

Based on our *in vitro* results showing higher binding for avelumab to PD-L1 in IFN-γ-stimulated NCIN87 gastric cancer cells compared to atezolizumab (**Figures 2A, B**), we conducted PET imaging and biodistribution analyses of these two antibodies in NCIN87 gastric xenografts. In this study, *nu/nu* mice bearing NCIN87 gastric tumors were imaged with PET/CT using radiolabeled [^89^Zr]Zr-DFO-avelumab or [^89^Zr]Zr-DFO-atezolizumab. We added unlabeled antibody to the tracer at the moment of injection (21–23 µg of [^89^Zr]Zr-DFO-avelumab plus 27–29 µg of unlabeled avelumab) to improve tumor uptake with a prior specific antibody dose loading (28). Tumor uptake of both radiotracers was assessed at 24, 48, and 72 h post-injection of the radiolabeled antibodies (**Figure 2C**, **Supplementary Figures 7, 8**). The PET images showed a more favorable biodistribution of [^89^Zr]Zr-DFO-avelumab *in vivo*, with higher binding uptake in NCIN87 gastric tumors compared to [^89^Zr]Zr-DFO-atezolizumab across all time-points.

Consistent with the PET imaging findings, we investigated the biodistribution in *ex vivo* organs. [^89^Zr]Zr-DFO-avelumab had higher tumor-to-organ ratios in all major organs than [^89^Zr]Zr-DFO-atezolizumab (**Figures 2D, E**, **Supplementary Figures 7, 8**). [^89^Zr]Zr-DFO-avelumab accumulated in NCIN87 tumors from 24 h (4.9% ID/g) to 48 h (6.4% ID/g) with a 42% loss occurring at 72 h (3.7% ID/g) compared to 24 h (**Supplementary Figure 7**). The uptake of [^89^Zr]Zr-DFO-avelumab and [^89^Zr]Zr-DFO-atezolizumab in NCIN87 tumors was higher when compared to that of a nonspecific [89Zr]Zr-DFO-IgG (~ 1.5% ID/g) (29). At 24 h post-injection, the overall tumor uptake (4.9% ID/g) of avelumab was lower compared to the blood pool (8.2% ID/g), with a subsequent 1.3-fold increase in tumor uptake and decrease in the blood observed at 48 and 72 h post-injection (**Supplementary Figure 7**). In contrast, for atezolizumab, the blood pool remained higher compared to tumor uptake from 24 to 72 hours post-injection (**Supplementary Figure 7**).

**Figure 3:**
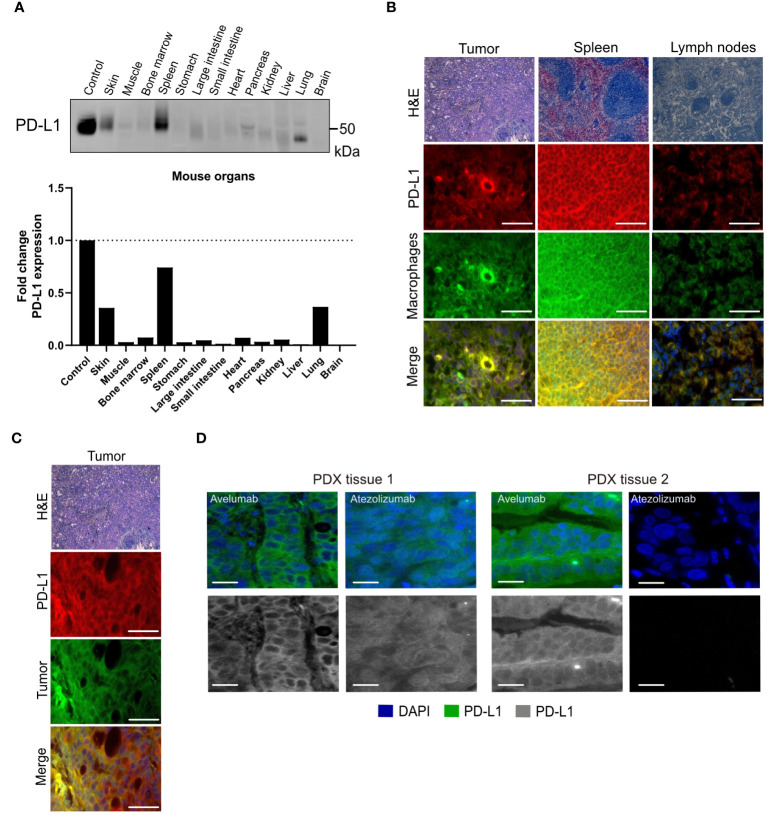
PD-L1 expression in cancer cells and mouse organs. **(A)** PD-L1 protein levels in mouse organs. **(B, C)** Hematoxylin and eosin (H&E) and PD-L1, macrophage (CD11b), tumor (cytokeratin) staining of NCIN87 tumors, murine lymph nodes, and murine spleen. Scale bar, 50 µm. **(D)** PD-L1 immunofluorescence staining in gastric tumor samples from two distinct tissues of patient-derived xenografts (PDX tissue 1 and PDX tissue 2) displaying higher staining intensity for the fluorescently labeled anti-PD-L1 antibody avelumab when compared with fluorescently labeled atezolizumab (green), nuclei stained with DAPI (blue). Bar, 50 µm.

Non-tumor tissue uptakes for avelumab were observed in the spleen (**Supplementary Figure 8**) and lymph nodes (**Supplementary Figure 9**), indicating the presence of PD-L1-expressing cells in these organs. These results are expected since avelumab and atezolizumab recognize both murine PD-L1 as well as human PD-L1. Additional Western blot analyses demonstrated PD-L1 expression in mouse organs, including high levels in the spleen (**Figure 3A**, **Supplementary Figure 10**). PD-L1 expression extends to macrophages, select activated T cells and B cells, dendritic cells, and certain epithelial cells (30). Although we utilized immunocompromised mice in these studies, it is important to note that these mice have B-cells, functional macrophages, and functional NK cells. Thus, the interaction of PD-L1 targeting antibodies with macrophages or other host immune cells will inevitably impact biodistribution (31, 32). Immunofluorescence staining of tissues excised from the mice following biodistribution studies showed that PD-L1 expression in the tumor, spleen, and lymph nodes co-localized with the macrophage marker CD11b (**Figure 3B**). Additionally, in the tumor, PD-L1 co-localization was observed with the pan-cytokeratin marker AE1/AE3 in NCIN87 tumor tissue (**Figure 3C**).

The lower avelumab uptake in the NCIN87 tumors when compared with spleen and lymph nodes results from the low to moderate levels of PD-L1 (44,000 PD-L1/cell) in NCIN87 cancer cells as determined *in vitro* (**Figure 1**). Consequently, when [^89^Zr]Zr-DFO-avelumab was co-administered with a 25-fold excess of unlabeled avelumab, a notable blockade in the spleen and lymph nodes uptake was observed (**Supplementary Figure 11**). These observations are similar to previous reports (28). Bone uptake for [^89^Zr]Zr-DFO-avelumab increased 2.7-fold from 24 h (1.5% ID/g) to 72 h (3.9% ID/g). This uptake in the bone, particularly on immune cells within the bone marrow microenvironment, could be specific, as reported by others (12), considering the stability of [^89^Zr]Zr-DFO-avelumab in serum (**Supplementary Figure 6**). Additionally, uptake in the bone can be a result of metabolites accumulation in the bone. The liver uptake of [^89^Zr]Zr-DFO-avelumab probably reflects antibody metabolism and elimination (12).

To further determine differences in avelumab versus atezolizumab detection of PD-L1 in tissues of gastric patient-derived xenografts (PDX), we performed immunofluorescence analyses using fluorescently labeled avelumab or atezolizumab. Consistent with the results from PET imaging and biodistribution, the fluorescence intensity of PD-L1 was higher in samples incubated with fluorescently labeled avelumab compared to atezolizumab in two different gastric tumor samples (**Figure 3D**).

In summary, these observations suggest that avelumab accumulates in gastric tumors to enable PD-L1 PET imaging.

### 3.4 PD-L1 glycosylation in gastric cancer cells

**Figure 4:**
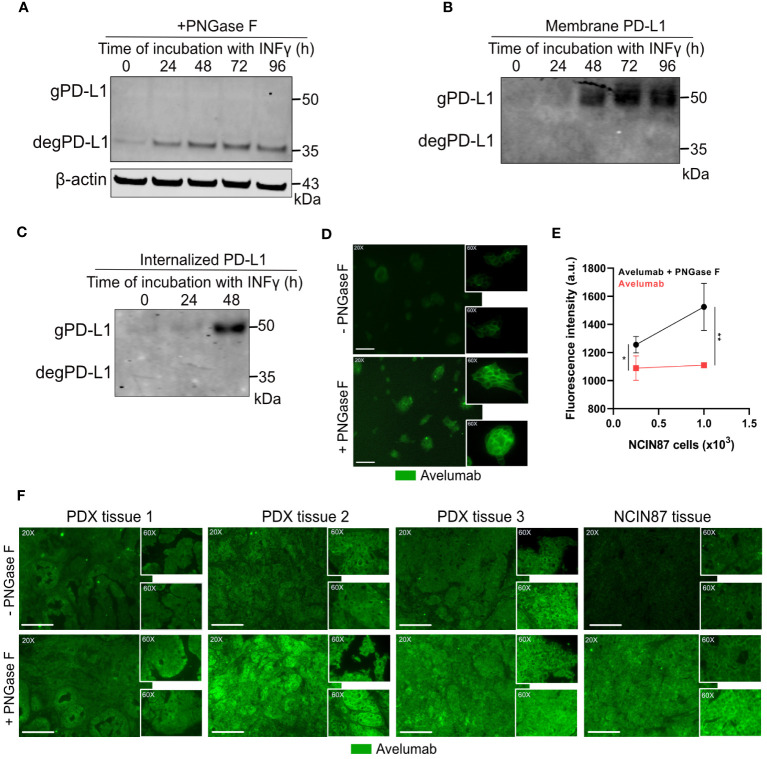
Avelumab binding is enhanced after deglycosylation of NCIN87 cancer cells by PNGase F treatment. **(A)** Glycosylation pattern of PD-L1 in NCIN87 gastric cancer cells. Cell lysates were treated with PNGase F and analyzed by Western blot. Glycosylated and deglycosylated PD-L1 are detected at around 50 kDa and 35 kDa, respectively. **(B, C)** Western blot of biotinylated cell surface-associated and internalized PD-L1 in NCIN87 cells. **(D, E)** Fluorescence intensity of fluorescently labeled avelumab in NCIN87 cells and gastric PDXs with and without PNGase F treatment. Scale bar, 50 µm. * p< 0.05, ** p< 0.01.

While performing Western blot analyses of PD-L1 protein levels in NCIN87 gastric cancer cells, we detected PD-L1 protein levels at approximately 50 kDa (**Figure 1A**). Previous studies have indicated that PD-L1 glycosylation often results in a heterogeneous pattern in expression on Western blots (19, 33). To further determine whether this pattern corresponds to PD-L1 glycosylation, we treated NCIN87 gastric cancer cells with recombinant glycosidase (peptide-*N*-glycosidase F; PNGase F). PNGase F eliminates the entire *N*-glycan structure. Western blot analyses of NCIN87 cancer cells treated with PNGase F demonstrated a significant reduction in a substantial portion of the 50 kDa PD-L1, now appearing at 35 kDa (**Figure 4A**). Expanding our investigations in NCIN87 gastric cancer cells to two different PDX gastric cancer samples, we observed bands on the Western blots corresponding to glycosylated PD-L1. Upon PNGase F treatment, deglycosylation was observed (**Supplementary Figure 12**).

Previous studies have shown that extensive glycosylation of PD-L1 impedes the recognition of polypeptide antigenic regions by anti-PD-L1 antibodies, making these regions less accessible for antibody binding (19). Since effective antibody binding relies on the availability of PD-L1 on the surface of cancer cells, we sought to investigate the glycosylation status of cell-surface localized PD-L1 on NCIN87 gastric cancer cells. To this end, we employed biotin-binding protein and avidin beads for the extraction of membrane proteins (**Figure 4B**). A subsequent Western blot confirmed PD-L1 expression around 50 kDa, demonstrating the exclusive presence of glycosylated PD-L1 on the cell surface of gastric cancer cells. Additionally, PD-L1 glycosylation was observed in the internalized pool at 48 h post-IFN-γ incubation (**Figure 4C**).

Overall, these results show that PD-L1 is glycosylated in NCIN87 cancer cells.

### 3.5 Removal of N-linked glycosylation enhances avelumab binding to PD-L1 in gastric cancer cells

To further investigate whether the *N*-linked glycan structure of PD-L1 poses a hindrance to avelumab binding, we subjected NCIN87 gastric cancer cells to pretreatment with or without PNGase F, followed by analysis using immunofluorescence microscopy. The fluorescence intensity of PD-L1 detected with avelumab was enhanced after PNGase F treatment compared to untreated cells (**Figure 4D**). These findings were further confirmed through a binding assay. As shown in **Figure 4E**, the addition of PNGase F increased avelumab binding to NCIN87 gastric cancer cells in a manner dependent on cell number. Our findings in gastric cancer cells were further validated in tissues from NCIN87 tumors and three distinct tissues of PDX samples (**Figure 4F**).

Our studies align with previous studies that have shown an enhancement in the anti-PD-L1 signal detected by FDA-approved PD-L1 antibodies following deglycosylation in cancer cells (**Supplementary Figure 13**) (19).

## 4 Discussion

Our preclinical investigations demonstrate the complexities of PD-L1 dynamic expression and heterogeneity that influence PD-L1 antibody binding and, consequently, their potential as PET imaging agents in gastric tumors. PET imaging with [^89^Zr]Zr-DFO-avelumab revealed intra-tumoral heterogeneity, emphasizing the spatial complexity of PD-L1 expression in gastric cancer (**Figure 1**). Notably, avelumab exhibited superior uptake to NCIN87 cells in both *in vitro* and *in vivo* investigations (**Figures 2**, **3**), underscoring its distinct characteristics compared to atezolizumab. Temporally regulated PD-L1 expression patterns were observed in Western blot analyses, with glycosylation further adding a layer of complexity to antibody binding. Removal of *N*-linked glycosylation enhanced avelumab binding to PD-L1 (**Figure 4**), suggesting the potential impact of glycosylation on antibody uptake. Collectively, our findings contribute to an understanding of PD-L1 in gastric cancer, crucial for advancing PET imaging approaches for successful clinical application.

Preclinical and clinical studies exploring PET imaging with PD-L1 targeting antibodies have shown the potential of this technology in tumor selection for immunotherapy (12, 15, 16). Nevertheless, the implementation of such a technique in gastric cancer requires the development of imaging probes and thorough validation processes. Our investigations in preclinical models of gastric cancer demonstrate that [^89^Zr]Zr-DFO-avelumab has a specific affinity for PD-L1 binding in NCIN87 cancer cells that express low to moderate levels of PD-L1. *In vivo*, [^89^Zr]Zr-DFO-avelumab has appropriate stability to be retained in NCIN87 tumors. Similar to previous reports, uptake was heterogeneous in NCIN87 tumors, varying from mouse to mouse (12).

Avelumab exhibits a superior binding affinity to PD-L1 compared to atezolizumab, as evidenced by the lower dissociation constant (K_d_) of avelumab relative to atezolizumab (26, 27). The low sub-nanomolar K_d_ value associated with avelumab suggests that [^89^Zr]Zr-DFO-avelumab can detect lower concentrations of PD-L1 compared to [^89^Zr]Zr-DFO-atezolizumab. This difference in K_d_ values aligns with previous research findings, which reported a 5-fold increase in the K_d_ value for radiolabeled atezolizumab when compared to avelumab (7, 28, 34). The superior binding affinity of avelumab may explain its enhanced uptake in our preclinical models of gastric cancer. In contrast to prior studies employing atezolizumab, where the specific activity was higher than the one used in this study (35), here we maintained it at the same level as previous investigations with avelumab (28). Future research will look into optimizing the specific activity of these radiolabeled antibodies for improved gastric cancer PET imaging.

PD-L1 undergoes *N*-glycosylation (20, 33) and this modification influences PD-L1 stabilization, antibody binding, and therapeutic efficacy (19). Notably, similar to previously reported studies, PD-L1 is mostly glycosylated in gastric cancer cells and PDX samples. Previous studies have shown that PD-L1 *N*-glycosylation is negatively associated with antibody recognition of PD-L1 in cancer cells (19). Our experiments, involving the removal of *N*-linked glycosylation, demonstrated a significant enhancement in avelumab’s binding to PD-L1. Considering the substantial glycosylation observed on cell-surface PD-L1 in gastric cancer cells, the generation of antibodies against glycosylated PD-L1 or the use of pharmacologic approaches for PD-L1 deglycosylation could hold the potential to enhance antibody binding. In the context of antibody-based imaging, these strategies would become particularly relevant as they could optimize the recognition and binding of antibodies to PD-L1.

Building upon our findings demonstrating the superior binding affinity of avelumab to PD-L1 compared to atezolizumab, our preclinical study aligns with others (28) suggesting that avelumab-PET may present an improvement over [^89^Zr]Zr-DFO-atezolizumab. However, the translation of these preclinical results into clinical applications requires a comprehensive consideration of *in vivo* pharmacokinetics of these radiolabeled monoclonal antibodies. Our investigations into the biodistribution of avelumab and atezolizumab, radiolabeled on the same day and administered at the same total mass of radiolabeled antibody with the addition of unlabeled antibody, demonstrated comparable distribution in non-tumor tissues, particularly in organs such as the spleen, liver, lymph nodes, and bone. While spleen and lymph node uptakes are attributed to specific PD-L1 expressing immune populations, liver uptake is more likely associated with the clearance of the radiolabeled antibody and its metabolites rather than a specific targeting (12, 36, 37). The uptake in the bone could either be specific because of antibody uptake in bone marrow over time or unspecific because of metabolites accumulation in the bone.

The complex pharmacokinetics of anti-PD-L1 antibodies, influenced by factors such as target interaction and immune responses, play a pivotal role in determining their *in vivo* biodistribution. Avelumab’s biologically intact Fc domain facilitates antibody-dependent cellular cytotoxicity by binding to Fc receptors on immune cells. In contrast to avelumab, atezolizumab lacks Fc receptor-mediated binding (25). The immunodeficiency status of the host mouse, PD-L1 expressing host cells, and other molecular properties unique to the antibody, including biological origin and glycosylation, further contribute to their differential *in vivo* behavior (38–40). Additionally, clinical data indicating avelumab’s faster clearance (41, 42) compared to atezolizumab underscores the potential for achieving suitable target-to-nontarget ratios earlier with [^89^Zr]Zr-DFO-avelumab, allowing for earlier acquisition of PET images. While promising clinical imaging results with antibody PD-L1 PET have been reported (12, 13), the optimization of PD-L1 immuno-PET agents and their inclusion in clinical trials are crucial steps to establish their predictive value as imaging agents. The challenges associated with the dynamic expression of PD-L1 in tumors and its expression beyond the tumor necessitate further development of small molecules or antibody fragments and their labeling with shorter-lived radionuclides (43, 44), such as fluorine-18, for clinical applications.

In summary, our study highlights the complexities in PD-L1 imaging, including PD-L1 dynamic expression, tumor heterogeneity, and glycosylation that affect antibody uptake in gastric tumors. These findings underscore the need for further research in radiotracer development with tumor specificity and the ability for multiple imaging time points, combined with a further understanding of PD-L1 biology and image analyses for successful PD-L1 imaging implementation in cancer.

## Data availability statement

The original contributions presented in the study are included in the article/**Supplementary Material**. Further inquiries can be directed to the corresponding author.

## Ethics statement

Ethical approval was not required for the studies on humans in accordance with the local legislation and institutional requirements because only commercially available established cell lines were used. The animal study was approved by Washington University School of Medicine’s Research Animal Resource Center and Institutional Animal Care and Use Committee. The study was conducted in accordance with the local legislation and institutional requirements.

## Author contributions

DI: Conceptualization, Data curation, Formal analysis, Investigation, Methodology, Validation, Visualization, Writing – original draft, Writing – review & editing. CS: Data curation, Formal analysis, Methodology, Visualization, Writing – review & editing. EB: Data curation, Methodology, Validation, Writing – review & editing, Formal analysis, Visualization. SS: Methodology, Writing – review & editing, Data curation, Validation. SP: Methodology, Writing – review & editing. AB: Writing – review & editing, Validation. RD: Writing – review & editing, Methodology. FD: Conceptualization, Writing – review & editing. HP: Conceptualization, Writing – review & editing. PP: Conceptualization, Data curation, Formal analysis, Funding acquisition, Investigation, Methodology, Project administration, Resources, Supervision, Validation, Visualization, Writing – original draft, Writing – review & editing.
