# Supplementary material for: PD-L1 has a heterogeneous and dynamic expression in gastric cancer with implications for immunoPET
Source: Front Immunol. 2024 Jun 10;15:1405485. doi: 10.3389/fimmu.2024.1405485 (PMC11194338; doi:10.3389/fimmu.2024.1405485)
Supplement: Supplementary file 1 [file DataSheet_1.docx]

PD-L1 has a heterogeneous and dynamic expression in gastric cancer with implications for immunoPET

**Dina Ibrahim^1,†^, Cristina Simó^1,†^, Emma L. Brown^1^, Shayla Shmuel^1^, Sandeep Surendra Panikar^1^, Alex Benton^1,2^, Rachel DeWeerd^2,3^, Farrokh Dehdashti^1^, Haeseong Park^3^, Patrícia M.R. Pereira^1,*^**

^1^Department of Radiology, Mallinckrodt Institute of Radiology, Washington University School of Medicine, St. Louis, MO 63110, USA

^2^Cancer Biology Graduate Program, Washington University School of Medicine

^3^Division of Biology and Biomedical Sciences, Washington University School of Medicine, St. Louis, MO 63110, USA

^4^Gastrointestinal Cancer Center, Center for Cancer Therapeutic Innovation, Dana-Farber Cancer Institute, Harvard Medical School

**^†^Equal contribution to this work**

***Correspondence:**Patrícia M. R. Pereira, PhD

ribeiropereirap@wustl.edu; P: +1 314-273-4898

Patrícia M. R. Pereira; ribeiropereirap@wustl.edu; P: +1 314-273-4898


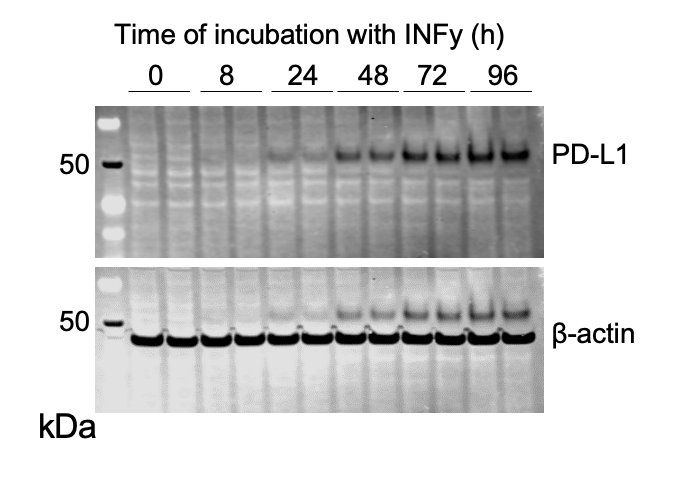


**Supplementary Figure 1.** Time-dependent expression of PD-L1 in NCIN87 cells incubated with INF-γ. Cell lysates of NCIN87 cells treated with INF-γ at different time points for 8, 24, 48, 72, and 96 h versus control and immunoblotting with anti-PD-L1 (E1L3N, Cell Signaling).

**
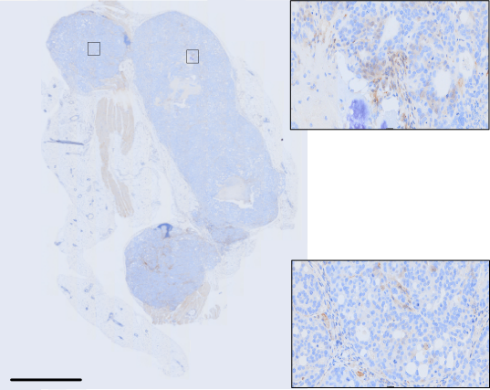
**

**Supplementary Figure 2.** Immunohistochemical (IHC) detection of PD-L1 in NCIN87 tumor tissue. Another representative image is shown in Figure 1b.


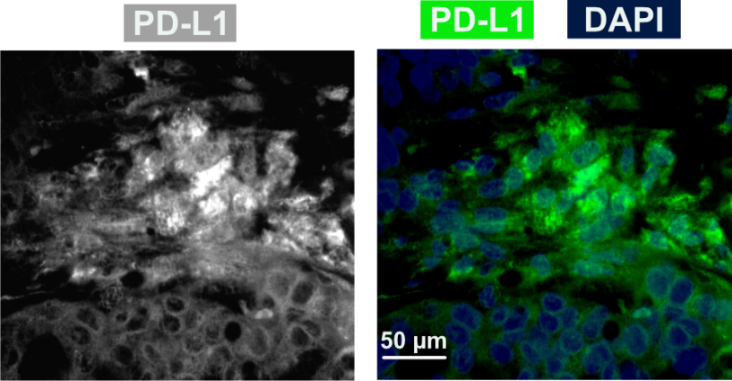


**Supplementary Figure 3.** PD-L1 immunofluorescence in NCIN87 tumors. Representative image of immunofluorescence staining of PD-L1 (green) and DAPI (blue) showing PD-L1 in NCIN87 tumors.


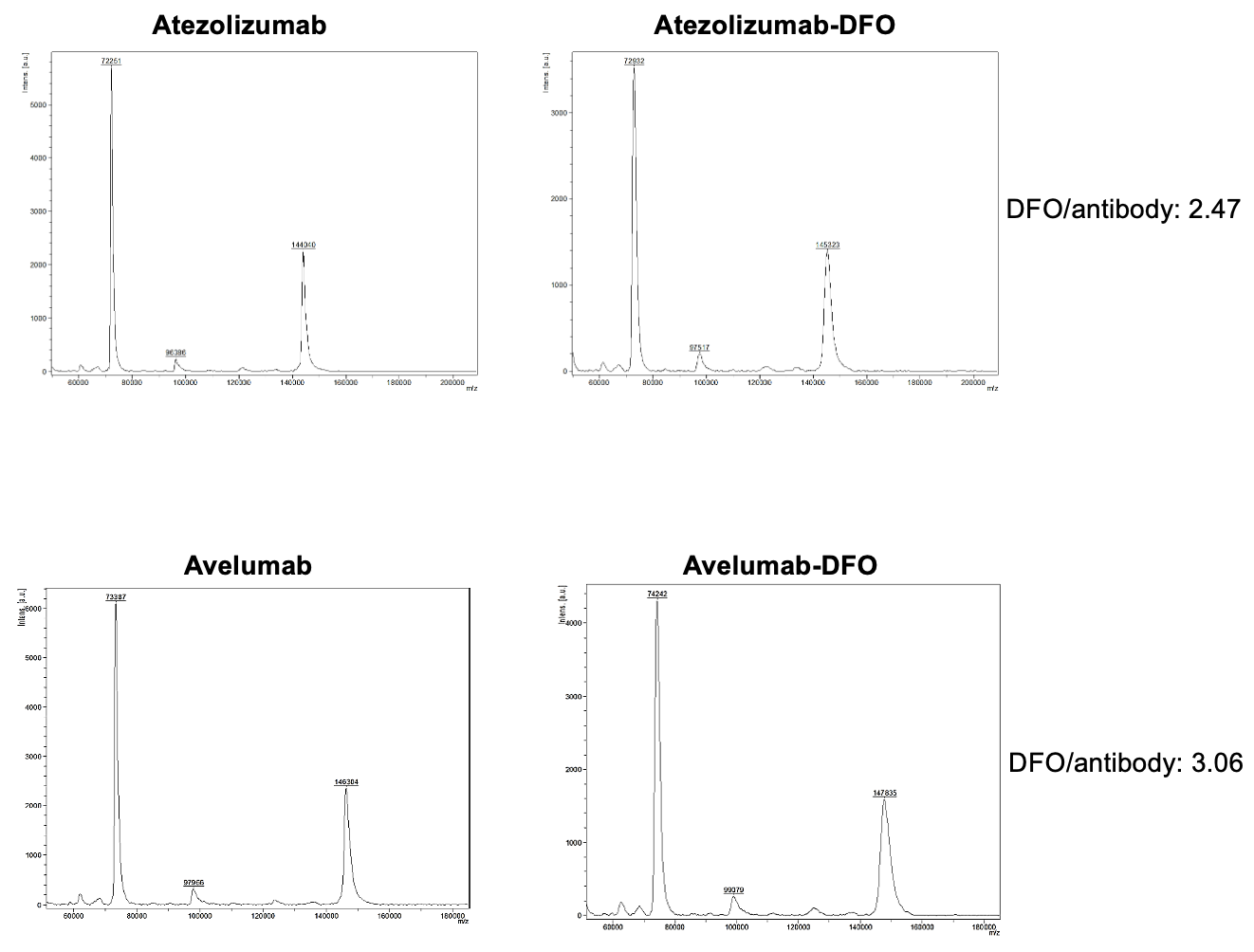


**Supplementary Figure 4.** MALDI spectra of native antibody (left panel) and DFO-conjugated avelumab or atezolizumab (right panel). The ratios 3.06 and 2.47 represent the average value of the DFO to avelumab or atezolizumab, respectively.


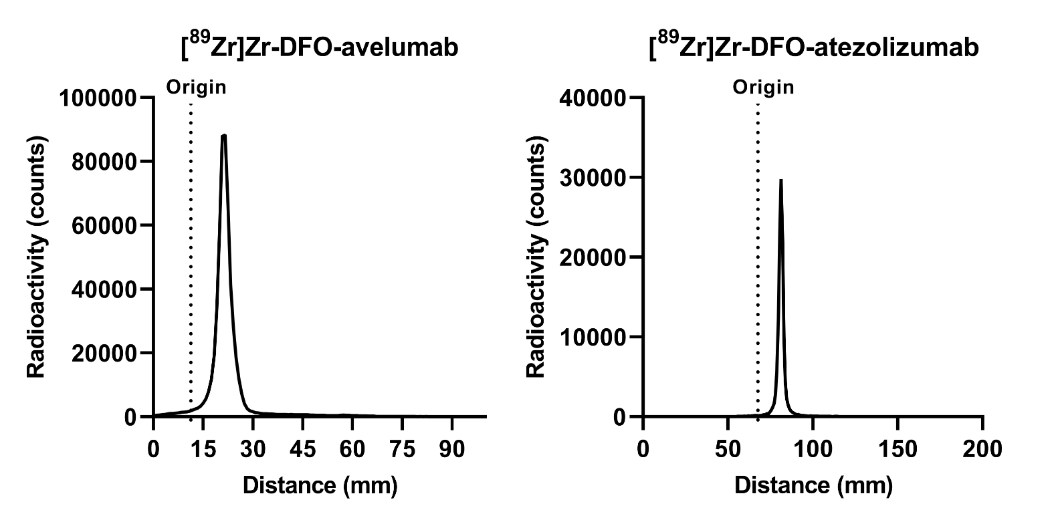


**Supplementary Figure 5.** Radio thin layer chromatography (radio-TLC) of [^89^Zr]Zr-DFO-avelumab and [^89^Zr]Zr-DFO-atezolizumab after PD10 purification. Radio-TLCs were run on 50 mM EDTA pH 5.0 as mobile phase.


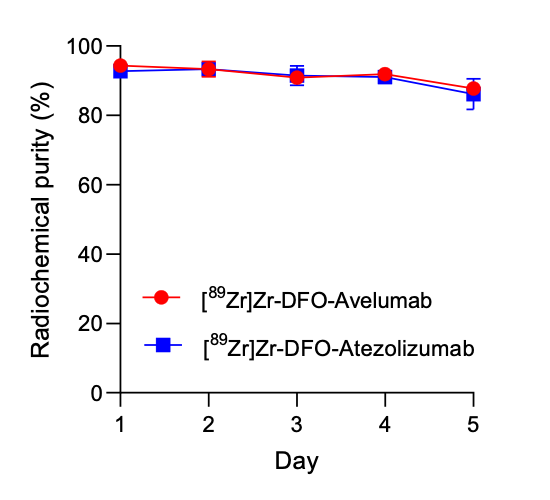


**Supplementary Figure 6.** Stability of [^89^Zr]Zr-DFO-avelumab and [^89^Zr]Zr-DFO-atezolizumab in human serum over time.


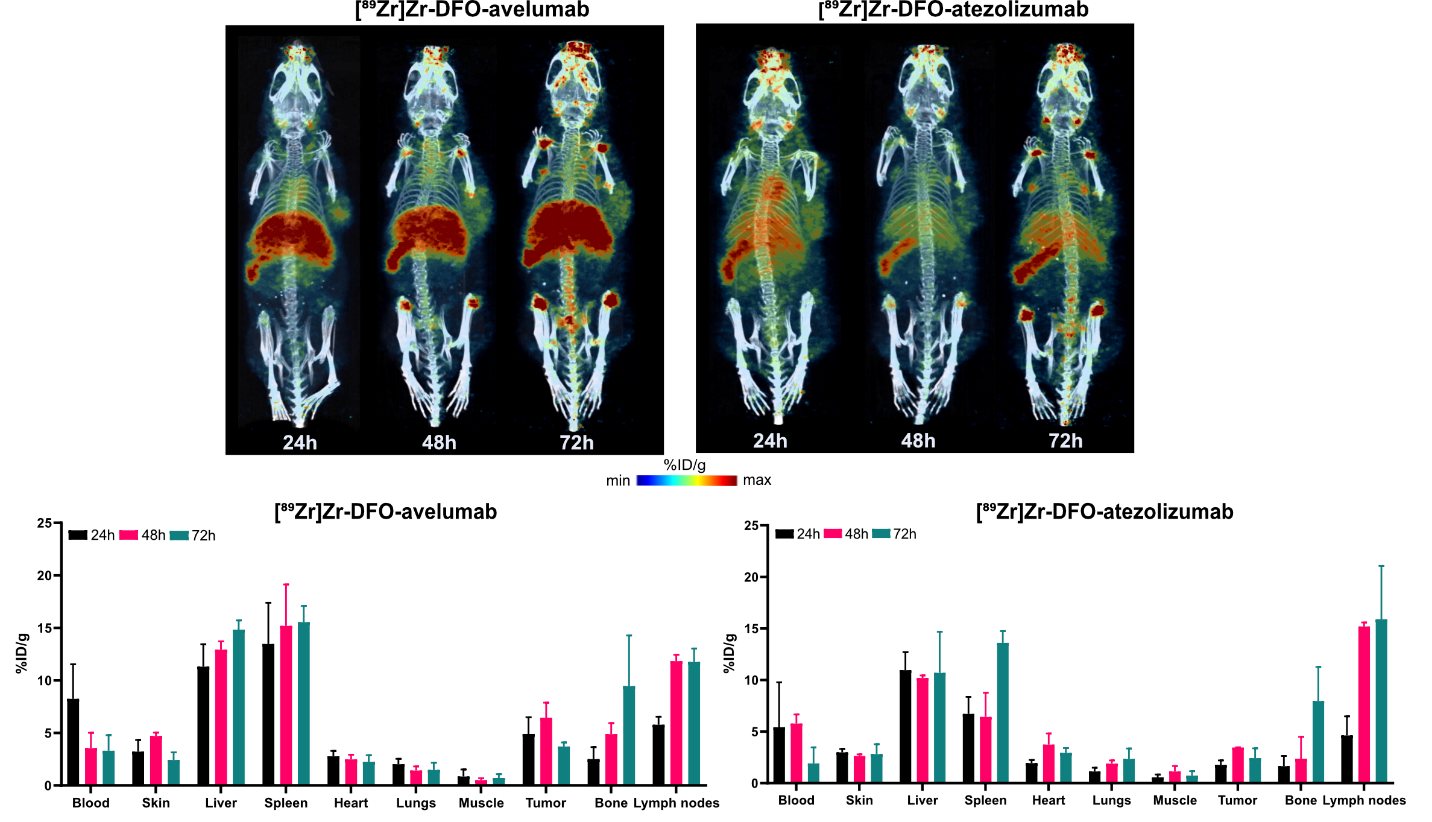


**Supplementary Figure 7.** PET/CT imaging and biodistribution of [^89^Zr]Zr-DFO-avelumab and [^89^Zr]Zr-DFO-atezolizumab in *nu/nu* mice bearing NCIN87 tumors. PET/CT images and biodistribution were collected at 24, 48, and 72 h post-injection of [^89^Zr]Zr-DFO-Avelumab (left) and [^89^Zr]Zr-DFO-Atezolizumab (right).


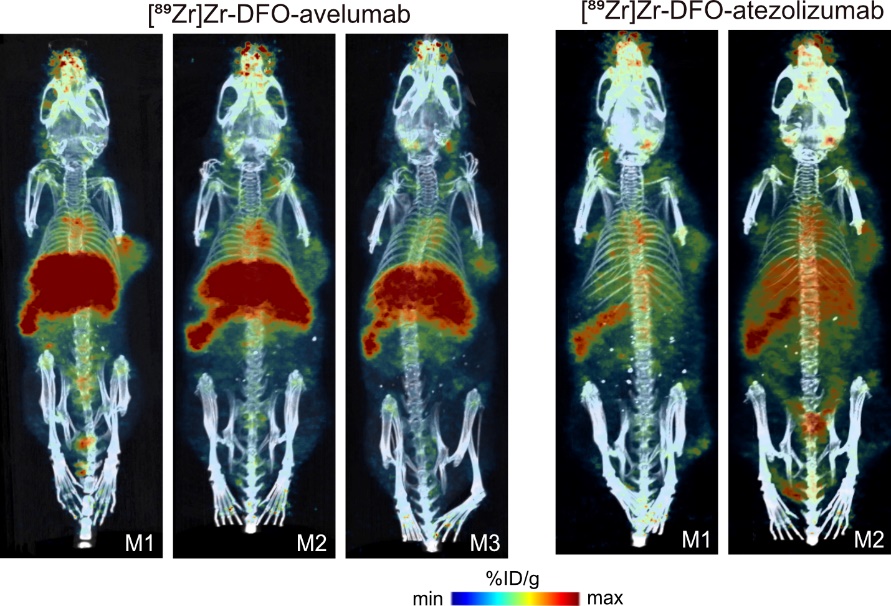


**Supplementary Figure 8.** PET images (maximum intensity projections, coronal view) of all the mice included in the study, obtained at 24 h post-injection of [^89^Zr]Zr-DFO-avelumab and [^89^Zr]Zr-DFO-atezolizumab. A representative image of each group is shown in Figure 2c.

**
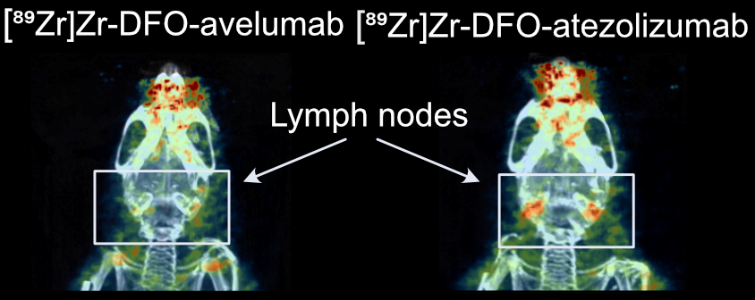
**

**Supplementary Figure 9.** Cropped PET images (maximum intensity projections, coronal view) showing lymph nodes uptake at 24 h post-injection of [^89^Zr]Zr-DFO-avelumab and [^89^Zr]Zr-DFO-atezolizumab.

**
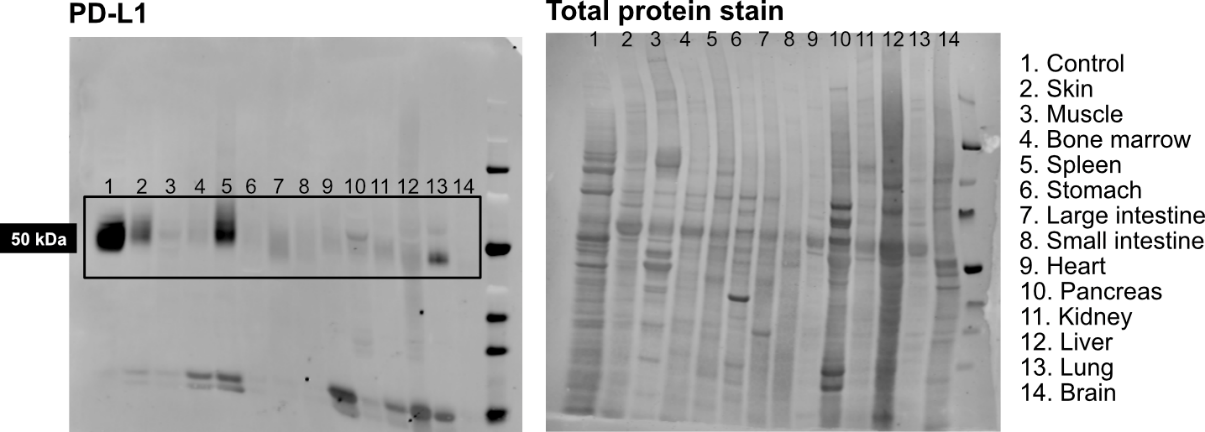
**

**Supplementary Figure 10.** Whole membrane of total protein for PD-L1 (left) and total protein stain (right) for mouse organs shown in Figure 3a.

**
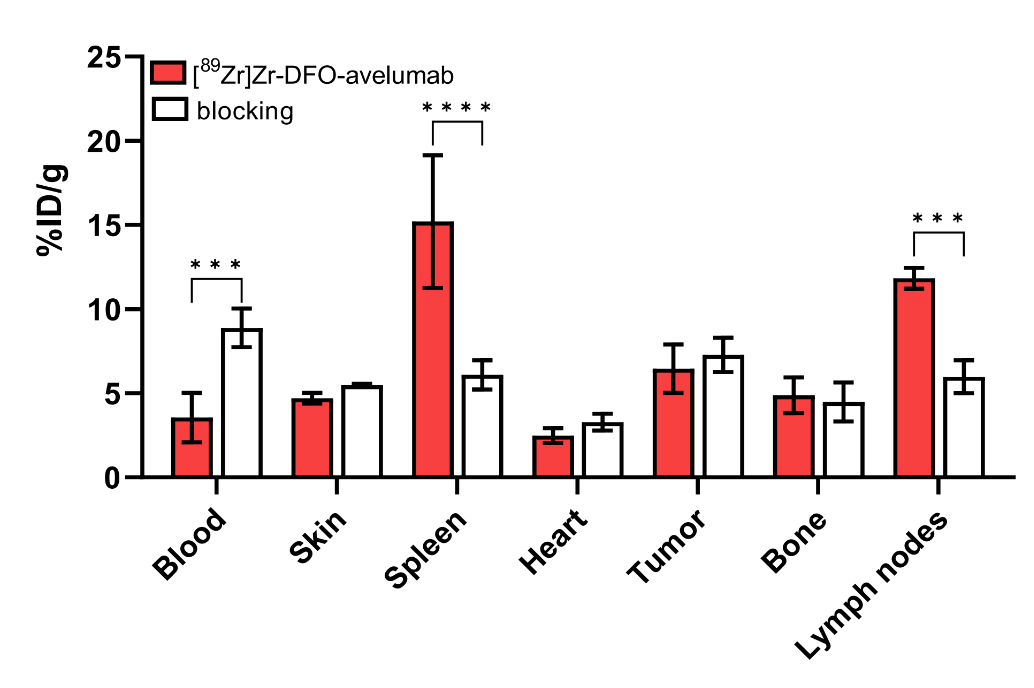
**

**Supplementary Figure 11.** *Ex vivo* biodistribution (%ID/g) at 48 h post-injection of [^89^Zr]Zr-DFO-avelumab (50 µg) and [^89^Zr]Zr-DFO-avelumab (50 µg) + unlabeled DFO-avelumab (25X, 1.25 mg) in NCIN87 xenografts. Statistical analyses were performed with two-way ANOVA: *** = p<0.001 **** = p<0.0001.

**
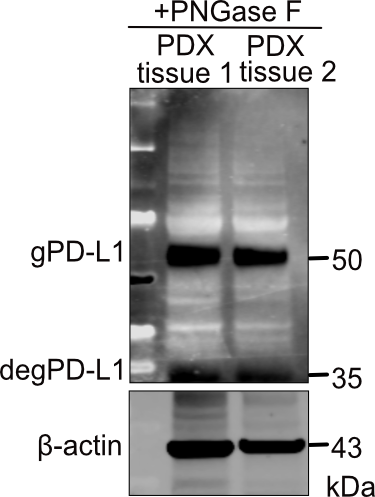
**

**Supplementary Figure 12.** Western blot images obtained for glycosylated (50 kDa) and deglycosylated (35 kDa) PD-L1 and β-actin in gastric PDX samples.


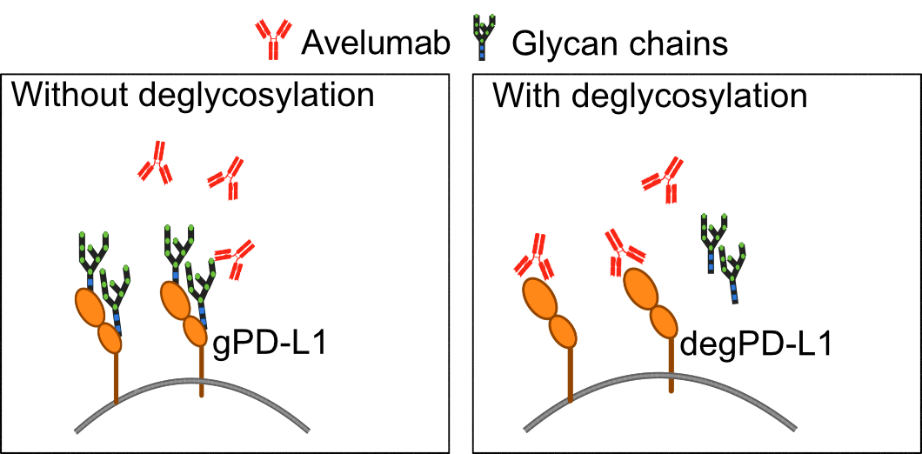


**Supplementary Figure 13.** Schematic representation of enhanced avelumab binding to gastric cancer cells after deglycosylation.
